# Supplementary material for: Online search trends and word-related emotional response during COVID-19 lockdown in Italy: a cross-sectional online study
Source: PeerJ. 2021 Aug 10;9:e11858. doi: 10.7717/peerj.11858 (PMC8362677; doi:10.7717/peerj.11858)
Supplement: Supplemental Information 1 [file peerj-09-11858-s001.docx]

**STROBE checklist**

| **Item** | **Item n.** | **STROBE Guidelines** | **Check** |
| --- | --- | --- | --- |
| **Title and abstract** | 1 | (a) Indicate the study’s design with a commonly used term in the title or the abstract | 🗹 |
|  |  | (b) Provide in the abstract an informative and balanced summary of what was done and what was found | 🗹 |
| **Introduction** |  |  |  |
| Background/rationale | 2 | Explain the scientific background and rationale for the investigation being reported | 🗹 |
| Objectives | 3 | State specific objectives, including any pre-specified hypotheses | 🗹 |
| **Methods** |  |  |  |
| Study design | 4 | Present key elements of study design early in the paper | 🗹 |
| Setting | 5 | Describe the setting, locations, and relevant dates, including periods of recruitment, exposure, follow-up, and data collection | 🗹 |
| Participants | 6 | (a) Cohort study—Give the eligibility criteria, and the sources and methods of selection of participants. Describe methods of follow-up | NA |
|  |  | Case-control study—Give the eligibility criteria, and the sources and methods of case ascertainment and control selection. Give the rationale for the choice of cases and controls | NA |
|  |  | Cross-sectional study—Give the eligibility criteria, and the sources and methods of selection of participants | 🗹 |
|  |  | (b) Cohort study—For matched studies, give matching criteria and number of exposed and unexposed | NA |
|  |  | Case-control study—For matched studies, give matching criteria and the number of controls per case | NA |
| Variables | 7 | Clearly define all outcomes, exposures, predictors, potential confounders, and effect modifiers. Give diagnostic criteria, if applicable | 🗹 |
| Data source /measurement | 8 | For each variable of interest, give sources of data and details of methods of assessment (measurement). | 🗹 |
|  |  | Describe comparability of assessment methods if there is more than one group | NA |
| Bias | 9 | Describe any efforts to address potential sources of bias | 🗹 |
| Study size | 10 | Explain how the study size was arrived at | 🗹 |
| Quantitative variables | 11 | Explain how quantitative variables were handled in the analyses. If applicable, describe which groupings were chosen, and why | 🗹 |
| Statistical methods | 12 | (a) Describe all statistical methods, including those used to control for confounding | 🗹 |
|  |  | (b) Describe any methods used to examine subgroups and interactions | 🗹 |
|  |  | (c) Explain how missing data were addressed | 🗹 |
|  |  | (d) Cohort study—If applicable, explain how loss to follow-up was addressed | NA |
|  |  | Case-control study—If applicable, explain how matching of cases and controls was addressed | NA |
|  |  | Cross-sectional study—If applicable, describe analytical methods taking account of sampling strategy | NA |
|  |  | (e) Describe any sensitivity analyses | NA |
| **Results** |  |  |  |
| Participants | 13 | (a) Report the numbers of individuals at each stage of the study—e.g., numbers potentially eligible, examined for eligibility, confirmed eligible, included in the study, completing follow-up, and analysed | 🗹 |
|  |  | (b) Give reasons for non-participation at each stage | NA |
|  |  | (c) Consider use of a flow diagram | NA |
| Descriptive data | 14 | (a) Give characteristics of study participants (e.g., demographic, clinical, social) and information on exposures and potential confounders | 🗹 |
|  |  | (b) Indicate the number of participants with missing data for each variable of interest | 🗹 |
|  |  | (c) Cohort study—Summarise follow-up time (e.g., average and total amount) | NA |
| Outcome data | 15 | Cohort study—Report numbers of outcome events or summary measures over time | NA |
|  |  | Case-control study—Report numbers in each exposure category, or summary measures of exposure | NA |
|  |  | Cross-sectional study—Report numbers of outcome events or summary measures | 🗹 |
| Main results | 16 | (a) Give unadjusted estimates and, if applicable, confounder-adjusted estimates and their precision (e.g., 95% confidence interval). | 🗹 |
|  |  | Make clear which confounders were adjusted for and why they were included | 🗹 |
|  |  | (b) Report category boundaries when continuous variables were categorized | NA |
|  |  | (c) If relevant, consider translating estimates of relative risk into absolute risk for a meaningful time period | NA |
| Other analyses | 17 | Report other analyses done—e.g., analyses of subgroups and interactions, and sensitivity analyses | 🗹 |
| **Discussion** |  |  |  |
| Key results | 18 | Summarise key results with reference to study objectives | 🗹 |
| Limitations | 19 | Discuss limitations of the study, taking into account sources of potential bias or imprecision. Discuss both direction and magnitude of any potential bias | 🗹 |
| Interpretation | 20 | Give a cautious overall interpretation of results considering objectives, limitations, multiplicity of analyses, results from similar studies, and other relevant evidence | 🗹 |
| Generalisability | 21 | Discuss the generalisability (external validity) of the study results | 🗹 |
| **Other information** |  |  |  |
| Funding | 22 | Give the source of funding and the role of the funders for the present study and, if applicable, for the original study on which the present article is based | 🗹 |

*Notes*: NA, not applicable.
